# Pharmacokinetics of Quercetin-Loaded Methoxy Poly(ethylene glycol)-b-poly(L-lactic acid) Micelle after Oral Administration in Rats

**DOI:** 10.1155/2017/1750895

**Published:** 2017-11-06

**Authors:** Li Lv, Chunxia Liu, Zhengrong Li, Fangming Song, Guocheng Li, Xingzhen Huang

**Affiliations:** ^1^School of Pharmacy, Guangxi Medical University, Nanning 530021, Guangxi, China; ^2^Department of Pharmacy, Sun Yat-sen Memorial Hospital, Sun Yat-sen University, Guangzhou, Guangdong 510120, China; ^3^Department of Pharmacy, Zengcheng District People's Hospital of Guangzhou, Guangzhou, Guangdong 511300, China; ^4^Department of Cell Biology, Nanjing Medical University, Nanjing, Jiangsu 211166, China

## Abstract

The purpose of this study was to evaluate the potential of micelle to change the pharmacokinetics of quercetin (QUT), with a primary goal of enhancing its oral bioavailability. QUT-loaded methoxy poly(ethylene glycol)-*b*-poly(L-lactic acid) micelle (QUT-loaded MPEG-*b*-PLLA micelle) was prepared by a thin-film hydration method, resulting in a particle size of 88.5 nm. A liquid chromatography tandem-mass spectrometry (LC-MS/MS) method was developed and validated for determination of QUT in rat plasma. The chromatographic separation was performed on an Agilent Eclipse-C_18_ (4.6 mm × 50 mm, 3.5 *μ*m) with an isocratic mobile phase system consisting of water and methanol (30 : 70, *v*/*v*) at a flow rate of 0.4 mL/min. Calibration curves were linear over the concentration ranges of 2.5–2000 ng/mL for QUT. The micelle was orally administered at a single does in rats, and the pharmacokinetic parameters were evaluated and compared with that administered with the QUT aqueous suspension. The results show that the micelle was able to increase the QUT's oral bioavailability 9-fold compared to the QUT aqueous suspension. These results suggest that methoxy poly(ethylene glycol)-*b*-poly(L-lactic acid) is a potential carrier for the oral delivery of QUT.

## 1. Introduction

Quercetin (QUT) is a flavonoid found in onions, apples, tea, and some vegetables [1]. It has been reported to be strong antioxidant because of its ability to scavenge free radicals and strong anti-inflammatory due to its inhibition of the proinflammatory cytokine TNF-*α* [2, 3]. Apart from these pharmacological activities, QUT has also shown strong antiproliferative, antiviral, and neuroprotection activities [1, 4]. In spite of these beneficial pharmacological properties, QUT's application* in vivo* is hampered by its low aqueous solubility and poor stability. The oral bioavailability of QUT is <17% in rats and ~1% in humans [5, 6]; this low bioavailability would be a consequence of QUT's lipophilic character. QUT which exhibited poor water solubility, low permeation, and short biological half-life was classified as a class IV compound according to the Biopharmaceutical Classification System [7, 8]. Therefore, there is a need to improve the oral bioavailability of QUT in the further study and application of QUT.

In recent years, more attention has been paid to polymeric micelles due to their ability to solve the problems of drugs that were associated with poor water solubility and low oral bioavailability [9–13]. Polymeric micelles formed by self-assembly of amphiphilic copolymers were considered as one of the most effective strategies to render the hydrophobic drug dispersible in aqueous solution. Moreover, amphiphilic copolymers have a lower critical micelle concentration and are more stable than conventional low-molecular-weight surfactants [14]. Many studies have shown that polymeric micelles have the ability to improve solubility, oral absorption, bioavailability, and pharmacological effect of hydrophobic drug [10, 15, 16]. Methoxy poly(ethylene glycol)-*b*-poly(L-lactic acid) (MPEG-*b*-PLLA) is one of the amphiphilic copolymers and it could be used to form micelles to encapsulate many drugs [17, 18].

In this study, MPEG-*b*-PLLA was used as a carrier to prepare QUT-loaded MPEG-*b*-PLLA micelle. A bioanalytical method based on LC-MS/MS was developed and validated to determine QUT in rat plasma. The QUT-loaded MPEG-*b*-PLLA micelle was orally administered at a single dose in rats, and the pharmacokinetic parameters of QUT from the micelle were calculated and compared with those from QUT aqueous suspension.

## 2. Materials and Method

### 2.1. Materials

Quercetin and luteolin (internal standard, IS) were purchased from Aladdin (Shanghai, China). MPEG-*b*-PLLA was obtained from Jinan Daigang Biomaterial Co., Ltd. (Jinan, China). Methanol of HPLC grade was purchased from Anaqua Chemicals Supply (Wilmington, DE, USA). Ultrapure water was purified using a Millipore Milli-Q system (Millipore, Bedford, MA, USA).

### 2.2. Preparation of Drug-Loaded Micelle

The QUT-loaded MPEG-*b*-PLLA micelle was prepared by a thin-film hydration method [19]. In brief, MPEG-*b*-PLLA (90 mg) and quercetin (10 mg) were codissolved in 50 mL solutions containing methanol and chloroform (3/7, v/v). After sonication at room temperature for 0.25 h, the solvent was removed by rotary evaporation, and then a drug/MPEG-*b*-PLLA matrix was obtained. The matrix was further dried under high vacuum at 37°C overnight to form a dried solid film. Then the film was hydrated with 20 mL of ultrapure water using a probe-type sonicator (Xin Zhi Biotechnology Co., Ltd., China) at 200 w for 5 min to form a solution. The resulting solution was filtered through a 0.22 *μ*m membrane filter to remove any aggregated particles and unencapsulated drug. After filtration, the QUT-loaded MPEG-*b*-PLLA micelle solution was obtained and kept at 4°C for further use.

### 2.3. Particle Size and Zeta Potential Measurement

The mean particle size and polydispersity index were measured by dynamic light scattering (DLS) using a Zeta Size Nano-S (Malvern Instrument) at a detection angle of 173°C. The zeta potential was determined by the light scattering method using 90 Plus Particle Size Analyzer (BC Haven Instruments Corporation). All measurements were repeated three times at the temperature of 25°C.

### 2.4. Measurement of Encapsulation Efficiency (EE) and Drug Loading Content (DL)

EE and DL of the QUT-loaded MPEG-*b*-PLLA micelle were determined by the previously reported method [20]. A Shimadzu HPLC system equipped with a SPD-10 Avp detector, a LC-10ADvp pump, and a Diamonsil C_18_ reversed phase column (4.6 mm × 250 mm, 5 *μ*m) was used for the determination of QUT. The mobile phase was composed of a mixture of acetonitrile, 10 mM ammonium acetate buffer, and methanol (32/48/20, v/v/v), pumped at a flow rate of 1.0 mL/min. The amount of QUT encapsulated into the micelle was determined directly after dissolution of micelle in acetonitrile. After the appropriate dilutions in acetonitrile and filtration with a 0.22 *μ*m membrane filter, 20 *μ*L of the sample was injected into the HPLC system and the detection wavelength was set at 370 nm. The EE% was estimated by comparing the weight of QUT extracted from micelle with the feeding QUT. The DL% was estimated by comparing the weight of QUT extracted from micelle with the weight of the micelle.

### 2.5. *In Vitro* Drug Release Study

The release of QUT from micelle was studied by a dialysis method as previously described [20]. Briefly, 1 mL of QUT-loaded MPEG-*b*-PLLA micelle solutions was placed in dialysis bags with a molecular weight cut-off of 3500 Da (Snakeskin, Pierce, USA). The dialysis bag was suspended in a tube and then 30 mL of the release medium (consisting of PBS (pH 7.4) and 0.5% (v/v) of Tween) was added to the tube. The tubes were placed in a shaking water bath at 37°C, 120 rpm. At predetermined time intervals, the release medium in the tube was completely drawn and replaced with fresh release medium. After dilution with acetonitrile, the amount of QUT in the release medium was determined by HPLC method as described above.

### 2.6. Chromatograph System and Conditions for QUT Quantitation in Plasma

The triple quadrupole LC-MS/MS system consisted of a 1200 series HPLC system (Agilent Technologies, USA) and a mass spectrometer (6420 triple Quad LC/MS, Agilent Technologies, USA). Chromatographic separation was achieved on an Agilent Eclipse-C_18_ (4.6 mm × 50 mm, 3.5 *μ*m) column at 20°C with an isocratic mobile phase system consisting of water and methanol (30 : 70, v/v). The injection volume was 5 *μ*L and the flow rate was 0.4 mL/min. QUT and IS were all ionized by ESI source in negative ion mode. The MS parameters were as follows: capillary, 4000 V; gas temperature, 300°C; gas flow, 11 L/min; and nebulizer, 15 psi. Quantification was performed using multiple reaction monitoring (MRM) of the transition of* m/z* 301.1 → 151.0 with collision energy (CE) of 16 eV for QUT, and* m/z* 285.0 → 133.0 with CE of 32 eV for IS. The fragmentor voltage was kept at 135 and 160 V for QUT and IS, respectively. The system control and data analysis were performed by Mass Hunter Work station Software Qualitative Analysis (Version B.06.00) and Quantitative Analysis (Version B.05.02).

### 2.7. Preparation of Standard Solutions, Calibration, and Quality Control Samples

Stock solution of QUT (10 *μ*g/mL) and IS (60 *μ*g/mL) was prepared in methanol and stored at −20°C away from light. Subsequently, the working standard solutions of QUT were prepared by serial dilution of the stock solutions with methanol. Calibration standards solution were prepared daily using blank rat plasmas which were obtained from rats without administration of any drugs spiked with the appropriate working solution of QUT to yield the concentration of 2.5–2000 ng/mL. The quality control (QC) solutions were prepared at three concentrations of 7.5 (low concentration), 750 (medium concentration), and 1500 ng/mL (high concentration) by the similar method as that for the calibration standards solutions. All the calibration standards and QC solutions were stored at 4°C away from light and brought to room temperature before use. The experimental data were expressed as means ± standard deviation (SD) and all the experiments were done with six parallel samples.

### 2.8. Plasma Sample Preparation

50 *μ*L of blank rat plasma with 50 *μ*L of IS solution (2 *μ*g/mL) and 300 *μ*L of acetonitrile were placed in a 1.5 mL polypropylene microcentrifuge tube. The mixture was vertex for 0.5 min and then was centrifuged at 15,000 rpm for 10 min at 4°C. 5 *μ*L of supernatant was injected into the LC-MS/MS system for analysis.

### 2.9. Bioanalytical Method Validation

#### 2.9.1. Specificity

The specificity of the developed method was investigated by comparing chromatograms from six different rats' plasma samples with those of QC plasma samples and with the samples collected from rats after administration of QUT to find out interference from endogenous components.

#### 2.9.2. Linearity and Lower Limit of Quantification (LLOQ)

The linearity of the bioanalytical assay was determined by observed peak area ratios of analytes to IS (*Y*) versus the spiked concentrations of analytes (*X*) in the concentration range of 2.5–2000 ng/mL at least six-point calibration curves; the acceptance criterion for a calibration curve was a correlation coefficient (*r*) of 0.99 or better. The LLOQ was defined as the lowest concentration of the analytes in the calibration curve with acceptable precision within 20% and accuracy of 80–120%.

#### 2.9.3. Precision and Accuracy

Intraday and interday precision and accuracy were determined by analyzing the three different QC concentrations, six replicates at each concentration, on the same day and for three consecutive days. The assay accuracy was expressed as (measured concentration/added concentration) × 100%. The intra- and interday precision was expressed as RSD, and the accuracy was defined as the RE.

#### 2.9.4. Extraction Recoveries and Matrix Effect

The extraction recoveries of QUT were determined by comparing the peak areas from blank plasma samples spiked with QC working solutions before extraction with those from blank plasma samples spiked after extraction. The matrix effects were evaluated by comparing the peak areas of QUT from blank plasma samples spiked with QC working solutions before extraction with those QUT spiked in mobile phase at corresponding concentrations. The experiments were done with six parallel samples.

#### 2.9.5. Stability

The stability of the analytes in plasma was investigated under the following conditions: the long-term stability was evaluated by determining QC plasma samples stored at −20°C for 30 days; the freeze-thaw stability was determined after three freeze-thaw cycles; short-term stability was evaluated after the exposure of QC samples to room temperature for 24 h. They were investigated by determining QC plasma samples of the three concentration levels, six replicates at each concentration, and considered stable when 85–115% of the initial concentrations were got.

### 2.10. Pharmacokinetic Study in Rats

Healthy male SD rats weighing from 200 to 250 g were used for this PK study. The experiments were performed according to institutional guidelines of the University Committee on Use and Care of Animals, Guangxi Medical University. Twelve rats were randomly divided into two groups (six rats per group) for oral administration of a single dose of QUT (40 mg/kg) or QUT-loaded MPEG-b-PLLA micelle (equivalent to 40 mg/kg of QUT), respectively. The rats were fasted with free access to water overnight prior to the experiments. Blood sample (approximately 0.2 mL) was collected via the eye ground veins into microcentrifuged tubes containing 10 *μ*L of 15% K_2_EDTA solution at 0 (before drug), 0.083 h, 0.25 h, 0.5 h, 1 h, 2 h, 4 h, 6 h, 8 h, 10 h, 12 h, and 24 h after dose, according to the previous reported methods with some modifications [21–26]. The collected blood samples were immediately centrifuged at 5000 rpm for 10 min. The supernatant was transferred to tightly seal plastic tubes and stored at −20°C until analysis by LC-MS/MS.

### 2.11. Statistical Analysis

The pharmacokinetic parameters were calculated by the PKSolver software package (version 2.0, China Pharmaceutical University, Nanjing, China). Significant differences between group values were analyzed using one-tailed Student's *t*-test. Differences were considered statistically significant at *p* < 0.05.

## 3. Results and Discussion

### 3.1. Preparation and Characterization of the QUT-Loaded MPEG-b-PLLA Micelle

The prepared QUT-loaded MPEG-b-PLLA micelle was successfully obtained by a thin-film hydration method. The particle size of the micelle was 88.5 ± 2.6 nm with polydispersity index (PDI) of 0.13 ± 0.04, as assessed by dynamic light scattering (DLS). These results indicated that the prepared micelle presented monodisperse profile and narrow size distribution. The drug loading content and encapsulation efficiency of the prepared micelle were determined by HPLC method. The drug loading contents of the prepared micelle were 6.1 ± 0.4% and the encapsulation efficiency was 82.5 ± 2.1%. The method used for prepared micelle resulted in significant enclosure of QUT, and the process was found to be highly reproducible. The surface zeta potential of the prepared micelle was −8.72 ± 1.03 mV, indicating that the surface charge of the prepared micelle was negative.

**Figure 1:**
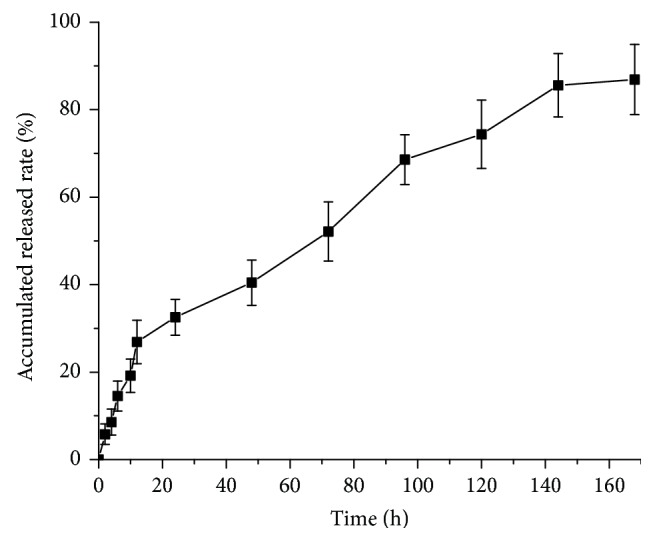
*In vitro* release profile of quercetin from the QUT-loaded MPEG-*b*-PLLA micelle in PBS (pH 7.4) at 37°C. Values reported as the mean ± SD (*n* = 3).

The* in vitro* release of QUT from micelle was examined simulating physiological conditions (37°C, PBS buffer pH 7.4). As shown in Figure 1, the QUT-loaded MPEG-*b*-PLLA micelle displayed slow and sustained release patterns, under physiological conditions. After the initial burst release over about 12 h, the release rate of QUT slowed down to show sustained release patterns. During the first 12 h, the percentages of QUT released from the QUT-loaded MPEG-*b*-PLLA micelle were 26.89 ± 1.99%. This initial burst release may be attributed to QUT desorption from the particle surface. After 168 h, approximately 86.89 ± 3.02% of the total QUT was found to be released from QUT-loaded MPEG-*b*-PLLA micelle. This sustained drug release profile could be characterized by the QUT diffusion through the polymeric matrix and subsequent diffusion/erosion of the polymeric matrix.

### 3.2. Bioanalytical Method Development

**Figure 2:**
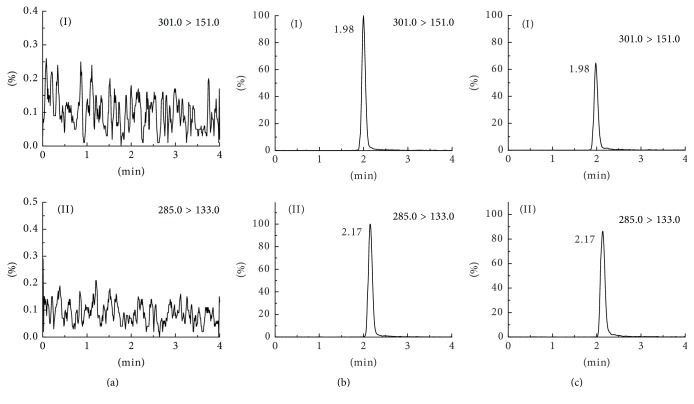
Representative LC-MS/MS chromatograms of quercetin (I) and IS (II) in rat plasmas: (a) a blank rat plasma sample; (b) a blank rat plasma sample spiked with quercetin (150 ng/mL), and IS (2 *μ*g/mL); (c) a plasma sample from a rat 6 h after an oral administration of QUT-loaded MPEG-*b*-PLLA micelle (the dose of QUT was 40 mg/kg).

A LC-MS/MS method for the determination of QUT in rat plasma has been developed and validated. Luteolin has similar structure with QUT, so it was used as internal standard (IS) in the development of the LC-MS/MS method. The automatic tuning mode was used to optimize MS conditions for detection of QUT and IS. Both positive and negative ion models were used and the results showed that QUT and IS showed higher responses in negative ion detection model than in positive model. As shown in Figure 2, the ion transitions of* m/z* 301.0 → 151.0 for QUT and* m/z* 285.0 → 133.0 for IS were used for multiple reaction monitoring (MRM). Isocratic elution was used to separate analytes. Chromatographic peaks of QUT and IS were sharp and baseline separation showed no interference with substances. The retention times were about 1.98 min and 2.17 min for QUT and IS, respectively. A simple and rapid protein precipitation method with acetonitrile was utilized to extract QUT and IS from the rat plasma sample and the results showed that protein precipitation with acetonitrile provided a higher recovery for both QUT and IS.

### 3.3. Bioanalytical Method Validation

#### 3.3.1. Specificity

The specificity of the developed method in this study was evaluated by comparing the chromatograms of QUT and IS in plasma and those of potentially interfering plasma components. Figure 2 shows the representative chromatograms obtained from a blank plasma sample (Figure 2(a)), plasma containing QUT (Figure 2(b)), and a plasma sample obtained 6 h after the oral administration of 40 mg/kg of QUT-loaded MPEG-*b*-PLLA micelle (Figure 2(c)). No interference from the endogenous compound with the QUT and the IS was detected under the described chromatographic conditions. These results demonstrated the high specificity of the LC-MS/MS method developed in this study.

#### 3.3.2. Linearity and LLOQ

The calibration curves were linear over the concentration range of 2.5–2000 ng/mL. Typical linear regression equation for the calibration curve was* Y* = 0.443466*X* + 0.018011 (*r* = 0.9990), where *Y* is the peak area ratio of QUT to the IS and* X* is the concentration of QUT (ng/mL). The correlation coefficients (*r*) of the calibration curves for QUT exceeded 0.99, indicating an excellent linearity over the concentration range. The lower limit of quantitation (LLOQ) was 2.5 ng/mL for QUT in rat plasma.

#### 3.3.3. Precision and Accuracy

The determined concentrations of QUT in plasma at three QC levels (7.5, 750, or 1500 ng/mL) and a summary of intra- and interday precision and accuracy for QUT was presented in Table 1. The RSD of each QC concentration examined was measured to be <7%, while the RE in the accuracy was within 7.5%. These dates were within the limits established by the FDA guidelines for the validation of bioanalytical methods. And these results indicated that the developed method is accurate and reliable.

#### 3.3.4. Extraction Recovery, Matrix Effect, and Stability

The mean extraction recoveries were 89.12 ± 4.58%, 102.39 ± 3.54%, and 96.68 ± 5.09% for QUT at 7.5, 750, and 1500 ng/mL, respectively. The mean extraction recovery of IS was 88.01 ± 5.09% at a concentration of 2 *μ*g/mL. These results indicated that the recoveries were consistent and reproducible and the protein precipitation method with acetonitrile was good for extracting QUT and IS in rat plasma. For matrix effect, they were 97.96 ± 2.63%, 104.44 ± 2.72%, and 95.90 ± 3.67 at 7.5, 750, and 1500 ng/mL, respectively, for QUT, and it was 96.73 ± 4.71% at 2 *μ*g/mL for IS. All of the matrix effect results were with the acceptable limits, suggesting that no obvious coeluting endogenous matrix influenced the ionization of QUT and IS in the plasma samples. As illustrated in Table 2, QUT was found to be stable in rat plasma under all testing conditions, including short-term storage, long-term storage, and freeze-thaw cycling.

### 3.4. Pharmacokinetic Study

**Figure 3:**
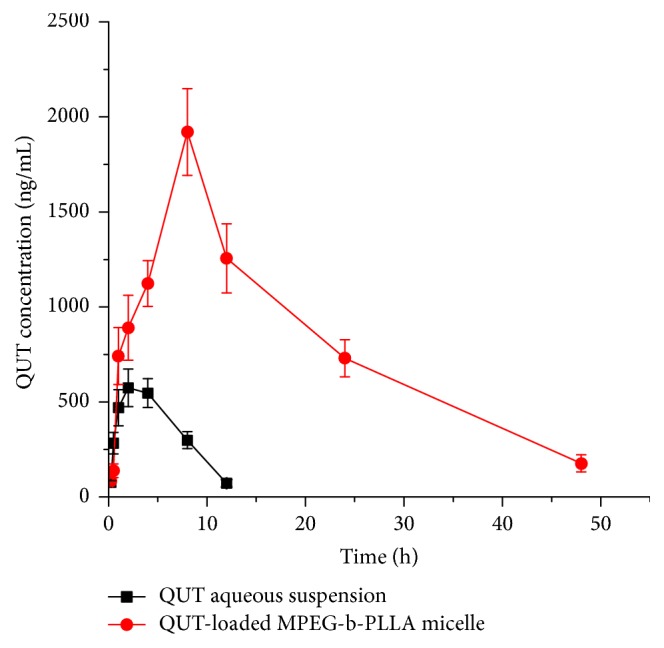
The* in vivo* plasma concentration versus time profiles of QUT after the oral administration of a single dose of QUT aqueous suspension or the QUT-loaded MPEG-*b*-PLLA micelle (*n* = 6).

The validated method was successfully applied to study the pharmacokinetic of QUT in rat plasma after an oral administration of QUT-loaded MPEG-b-PLLA micelle or QUT aqueous suspension with the same dose of QUT at 40 mg/kg. The mean plasma concentration-time profile is illustrated in Figure 3. The relevant pharmacokinetic parameters of QUT which were calculated using PKSolver software using the noncompartmental model were summarized in Table 3.

After the oral administration of a single dose of QUT aqueous suspension or the QUT-loaded MPEG-b-PLLA micelle, the maximum drug concentrations (*C*_max_) were 628.67 ± 64.66 ng/mL and 1920.83 ± 250.14 ng/mL for the QUT aqueous suspension and the QUT-loaded MPEG-*b*-PLLA micelle, respectively. Compared to QUT aqueous suspension, *C*_max_ of QUT from QUT-loaded MPEG-*b*-PLLA micelle was increased 3.1-fold. The increase in *C*_max_ indicates that the micelle was effective in increasing drug absorption. *T*_max_ of QUT was 3.0 ± 1.1 h and 7.3 ± 1.6 h in the QUT aqueous suspension treated and QUT-loaded MPEG-*b*-PLLA micelle treated rats, respectively. The delayed *T*_max_ of the micelle treated rats may be attributed to the sustained release of QUT from QUT-loaded MPEG-*b*-PLLA micelle. Furthermore, the results of MRT also confirm the sustained effect of QUT-loaded MPEG-*b*-PLLA micelle as compared with QUT aqueous solution, the MRT of QUT-loaded MPEG-*b*-PLLA micelle, and QUT aqueous solution were 20.2 ± 2.4 and 5.4 ± 0.5 h, respectively. The enhanced sustained effect of micelle may be partly due to the prolonged circulation of the micelle in the bloodstream. The clearance (CL) of QUT from the micelle was 2-fold lower than that of free QUT. The micelle decreased the QUT volume of distribution (*V*) by 9-fold compared to free QUT. The mean area under the curve (AUC_0–∞_) of QUT-loaded MPEG-*b*-PLLA micelle was 41677.10 ± 4573.95 h ng/mL, while the AUC_0–∞_ of orally administered QUT aqueous suspension was 4633.71 ± 557.67 h ng/mL, indicating that QUT-loaded MPEG-*b*-PLLA micelle provided a 9-fold increase in the relative oral bioavailability of QUT as compared with the QUT aqueous suspension. Our results demonstrated that MPEG-*b*-PLLA could serve as a carrier to increase the oral bioavailability of QUT. The enhanced bioavailability after oral administration of QUT-loaded MPEG-*b*-PLLA as compared with suspension, probably owing to increased solubility, absorption, and residence time of drug delivered.

## 4. Conclusion

In summary, QUT-loaded MPEG-*b*-PLLA micelle was successfully prepared by the thin-film hydration method. Besides, an analytical method for determining QUT in rat plasma was developed and validated. The developed and validated LC-MS/MS method was applied to study the pharmacokinetic study of QUT and the results demonstrated that most pharmacokinetic parameters of QUT were changed by the QUT-loaded MPEG-b-PLLA micelle. *C*_max_, *T*_max_, *t*_1/2_, AUC, and MRT of QUT were significantly increased, while CL and *V* were decreased by the QUT-loaded MPEG-*b*-PLLA micelle as compared with the QUT aqueous suspension. The QUT-loaded MPEG-*b*-PLLA micelle was able to increase the QUT bioavailability in 9-fold compared to the QUT aqueous suspension. It can be concluded that MPEG-*b*-PLLA could be a promising carrier for the oral delivery of QUT.

## Figures and Tables

**Table 1:** Accuracy and precision of LC-MS/MS method to determine QUT in rat plasma (in three validation days, six replicates at each concentration level per day).

Added concentration (ng/mL)	Measured concentration (ng/mL)	Precision (%, RSD)	Accuracy (%, RE)
Intraday			
7.5	7.37 ± 0.34	4.62	−1.74
750	743.33 ± 23.69	3.19	−0.98
1500	1607.67 ± 42.79	2.66	7.18
Interday			
7.5	7.50 ± 0.56	6.67	−0.05
750	747.89 ± 39.17	5.24	−0.28
1500	1592.44 ± 104.28	6.55	6.16

**Table 2:** Stability of QUT study under different storage conditions in rat plasma (*n* = 6).

Storage conditions	Concentration (ng/mL)		Precision (%, RSD)	Accuracy (%, RE)
	Added	Measured (mean ± SD)		
Three freeze-thaw cycles	7.5	7.37 ± 0.37	4.97	−1.75
	750	769.67 ± 26.96	3.60	2.62
	1500	1604.67 ± 72.33	4.51	6.98

−20°C for 30 days	7.5	7.76 ± 0.47	6.10	3.49
	750	776.17 ± 39.35	5.07	3.49
	1500	1616.33 ± 107.32	6.64	7.76

Room temperature for 24 h	7.5	7.39 ± 0.48	6.51	−1.49
	750	743.33 ± 32.96	4.43	−0.89
	1500	1590.00 ± 16.25	1.02	6.00

**Table 3:** Pharmacokinetic parameters of QUT after single oral administration of QUT aqueous suspension or QUT-loaded MPEG-*b*-PLLA micelle, in rat (*n* = 6).

Pharmacokinetic parameters	QUT aqueous suspension	QUT-loaded micelle
*T*_max_ (h)	3.0 ± 1.1	7.3 ± 1.6^#^
*t*_1/2_ (h)	2.9 ± 0.6	12.7 ± 2.3^#^
MRT_0–*∞*_ (h)	5.4 ± 0.5	20.2 ± 2.4^#^
*C*_max_ (ng/mL)	628.67 ± 64.66	1920.83 ± 250.14^#^
AUC_0–48_ (ng/mL*∗*h)	4318.42 ± 470.31	38312.97 ± 4346.72^#^
AUC_0–*∞*_ (ng/mL*∗*h)	4633.71 ± 557.67	41677.10 ± 4573.95^#^
CL (mL/h/kg)	36509 ± 6399	17730 ± 3387^#^
*V* (mL/kg)	8736 ± 1043	970 ± 115^#^

*T*_max_, time to reach maximum concentration; *t*_1/2_, plasma half-life; MRT, mean residence time; *C*_max_, maximum drug concentration; AUC, area under curve; CL: plasma clearance; *V*: apparent volume of distribution. ^ #^Pharmacokinetic parameters obtained with QUT-loaded micelle were significantly different from QUT aqueous suspension at *p* < 0.05.
